# Growth performance, reproductive parameters and fertility measures in young Nellore bulls with divergent feed efficiency

**DOI:** 10.1590/1984-3143-AR2022-0053

**Published:** 2022-10-24

**Authors:** Guilherme Fazan Rossi, Natália Marins Bastos, Dayane Priscila Vrisman, Naiara Nantes Rodrigues, Roberta Vantini, Joaquim Mansano Garcia, Erika Aline Ribeiro Dias, Flávia Fernanda Simili, André Lasmar Guimarães, Roberta Carrilho Canesin, Maria Eugênia Zerlotti Mercadante, Camila de Paula Freitas-Dell’Aqua, Flávia Regina Florencio de Athayde, Fabio Morato Monteiro, Gisele Zoccal Mingoti

**Affiliations:** 1 Faculdade de Ciências Agrárias e Veterinárias, Departamento de Radiologia, Reprodução e Saúde Única, Universidade Estadual Paulista, Jaboticabal, SP, Brasil; 2 Centro de Pesquisa de Bovinos de Corte, Instituto de Zootecnia, Sertãozinho, SP, Brasil; 3 Departamento de Reprodução Animal e Radiologia Veterinária, Faculdade de Medicina Veterinária e Zootecnia, Universidade Estadual Paulista, Botucatu, SP, Brasil; 4 Departamento de Produção e Saúde Animal, Faculdade de Medicina Veterinária, Universidade Estadual Paulista, Araçatuba, SP, Brasil

**Keywords:** bovine, embryo, male, residual feed intake, semen

## Abstract

The growth, sexual maturity and fertility-related parameters related of young Nellore bulls with divergent residual feed intake (RFI) raised on pasture were evaluated. After classification of 48 young males as low and high RFI (more and less efficient, respectively), the animals were evaluated for growth and reproductive parameters at 28-day intervals from 14.3 to 24.6 months of age. The semen was cryopreserved in the last sampling and fresh and post-thaw semen samples were evaluated. Low RFI bulls exhibited higher initial and final body weight (*P* < 0.05), but feed intake, body condition score and growth measures evaluated by carcass ultrasound were unaffected by RFI (*P* > 0.05). The scrotal circumference, sperm concentration, defects, and quality of fresh semen, and ultrasonographic testicular characteristics were unaffected by RFI (*P* > 0.05). However, velocity parameters such as average path and curvilinear velocities determined by computer-assisted sperm analysis of thawed semen submitted to the rapid thermoresistance test were improved (*P* < 0.05) in low RFI bulls, but this improvement in quality did not enhance *in vitro* sperm fertilizing ability. Our results demonstrated significant differences in metabolism and growth performance between bulls of divergent RFI. In addition, there was slight improvement in the semen quality of bulls with low RFI bulls, but this did not enhance *in vitro* fertilizing ability. Selection of beef bulls for RFI can be performed, which will result in economic benefits by improving the growth performance of the animals without affecting reproductive parameters.

## Introduction

Feeding costs represent a large part of the total cost of cattle production; thus, improving the feed efficiency of these animals may have economic benefits for livestock farming (Kennedy et al., 1993; Forbes, 2007). Within this context, the establishment of strategies designed to increase the efficiency of the herd is desirable in order to reduce feed intake as well as the environmental impact resulting from the methane emission of cattle (Herd et al., 2002).

Increasing attention has been given to the evaluation of the productive and reproductive performance of animals with divergent feed efficiency since the classification of animals according to residual feed intake (RFI) has been shown to be an important tool in the genetic improvement of beef cattle (Grion et al., 2014; Ceacero et al., 2016). The RFI is calculated as the difference between observed and expected intake in such a way that animals are classified as low RFI (more efficient: observed intake lower than predicted for observed gain) and high RFI (less efficient: observed intake higher than predicted) (Arthur et al., 2001; Crews, 2005; Mercadante and Grion, 2013; Berry and Crowley, 2013). In this way, the selection of animals classified as low RFI may result in offspring that consume less feed without comprising animal performance (Arthur and Herd, 2008), with economic benefits for pasture-based beef production systems (Herd et al., 2004).

Despite the economic benefits, some studies have reported an inverse relationship between male fertility and improved feed efficiency (Arthur et al., 2005; Basarab et al., 2007; Mu et al., 2016), such as worsening of sperm motility (Awda et al., 2013) and sperm morphology (Awda et al., 2013; Hafla et al., 2012). On the other hand, other studies found no changes in sperm quality of bulls selected for RFI (Fox et al., 2004). Therefore, there are conflicting reports in the literature on the impact of selecting animals with better feed efficiency (low RFI) on reproduction (Ferreira et al., 2018).

The aim of the present study was to compare pasture-raised young Nellore bulls classified as low and high RFI regarding their growth characteristics, sexual maturity and fertility-related parameters.

## Methods

### Animals and experimental design

All experimental procedures were approved by the Ethics Committee on Animal Experimentation of UNESP (Protocol No. 017361/13) and were conducted at the Centro de Pesquisa de Bovinos de Corte, Instituto de Zootecnia (IZ/APTA/SAA), Sertãozinho, São Paulo, Brazil.

Young Nellore (*Bos taurus indicus*) bulls were weaned at 7 months of age and were pre-selected to undergo feedlot performance testing to determine their RFI [*n* = 59 in the first year of the experiment and *n* = 71 (different animals) in the second year]. These animals were fed in a collective pen equipped with feeders of the GrowSafe® automatic feeding system (GrowSafe® Systems Ltd., Airdrie, Alberta, Canada) for at least 21 days for adaptation, followed by 98 days for individual feed intake recording. The animals were weighed every 14 days without fasting (Ceacero et al., 2016). The RFI was calculated for each bull as the error term of the equation proposed by Koch et al. (1963), in which DMI (average dry matter intake observed during the test) = β0 + βP*MBW + βG*ADG + ε(RFI), where β0 is the intercept of the equation, MBW is the metabolic body weight of the test, ADG is the average daily weight gain during the test, βP and βG are the regression coefficients of MBW and ADG, respectively, and ε is the error (i.e., RFI). After determining the RFI, the bulls sampled within the extreme classes of RFI were selected for the experiment, as follows: animals classified as low RFI [<0.5 standard deviation (SD) below the mean; *n* = 12 bulls per year of the experiment, totaling 24 bulls] and high RFI (>0.5 SD above the mean; *n* = 12 bulls per year, totaling 24 bulls). Thus, a total of 48 bulls were used in the two experimental groups of this study, being animals classified as low RFI (*n* = 24 bulls) and high RFI (*n* = 24 bulls), with a mean RFI of -0.86 ± 0.08 and 0.73 ± 0.08 kg DM/day, respectively. At the beginning of the experiment, the mean age of the animals was 14.3 ± 0.13 months and the mean weight was 389.5 ± 5.43 kg.

The experimental design was completely randomized with repeated measures over time. The experiment was carried out from December (year 1) to October of the next year (year 2; first replicate) and from December (year 2) to October of the next year (year 3; second replicate). The animals were evaluated for growth traits and reproductive parameters at 28-day intervals (from 14.3 ± 0.13 to 24.6 ± 0.13 months of age, totaling 12 evaluations per replicate), as detailed below.

### Management of animals on pasture

The bulls were kept on *Brachiaria brizantha* cv Marandu pasture with free access to clean water and mineral supplement (Raçaphós 80 FE STZ, Brasil Química Indústria e Com. Ltda, Batatais, Brazil). Animals were divided into two paddocks of 6 hectares (ha) with an initial stocking rate of 1.7 animal unit (AU)/ha (2.0 animals/ha), in a continuous stocking system at a fixed stocking rate. The volume of fecal excretion and indigestible neutral detergent fiber were evaluated using chromium oxide (Cr_2_O_3_) to estimate the feed intake of the animals, as described by Rossi et al. (2019) and following previously reported methods (Smith and Reid, 1955; Kimura and Miller, 1957; Detmann et al., 2001; Van Soest et al., 1991; Mertens, 2002; Detmann et al., 2012; Casali et al., 2008; Casali et al., 2009; Valente et al., 2011).

### Growth performance measures

The measures were taken at 28-day intervals, always by the same technician. The body condition score was rated on a scale from 1 (extremely lean) to 9 (extremely fat), as previously described (Wagner et al., 1988). The hip height measurement was performed by drawing a vertical line from the anterior portion of the sacrum to the ground with the aid of a metric tape measure.

### Ultrasound carcass evaluation

Backfat thickness, rib eye area and rump fat thickness were measured in bulls with the aid of an Aquila Vet real-time ultrasound scanner (Esaote Pie Medical, Pie Medical Equipment B.V., Maastricht, The Netherlands) equipped with a 170-mm long, 3.5-MHz linear transducer. Measurements were performed at 14.3 ± 0.09, 20.9 ± 0.09 and 24.6 ± 0.09 months of age, according to Herring et al. (1994) and following the steps described Rossi et al. (2019). The ultrasound scans were analyzed using the Echo Image Viewer 1.0 program (Pie Medical Equipment B.V. 1996).

### Blood sampling and analysis

Blood samples were collected into sterile tubes (Vacutainer^®^, Labnew^®^ Indústria e Comércio Ltda, Juiz de Fora, MG, Brazil) without anticoagulants or with EDTA or fluoride + EDTA by puncturing the coccygeal vein with a 21 G needle (Labor Import Comercial Imp. Exp. Ltd., Brazil). The tubes were centrifuged at 1500*g* at room temperature for 20 min. Plasma and serum were stored in microtubes and kept frozen at -20^o^C until the time of the assays.

Serum concentrations of total cholesterol and high-density lipoprotein (HDL) were measured using commercial diagnostic kits (Labtest Diagnóstica S.A., Brazil) according to the manufacturer’s recommendations. A Labquest® semi-automatic biochemical analyzer (Labtest, Minas Gerais, Brazil) was used to read the samples.

Plasma concentrations of testosterone (Coat-A-Count® Total Testosterone kit, Diagnostic Products Corporation, Los Angeles, CA, USA), insulin (Coat-A-Count Insulin kit, Diagnostic Products Corporation, Los Angeles, CA, USA) and leptin (Multi-species Leptin kit, Millipore, Billerica, MA, USA) were determined by radioimmunoassay using commercial kits according to the manufacturer’s recommendations. The sensitivity of the testosterone assay was 0.3 ng/mL and intra- and interassay coeffcients of variation (CVs) were 5.5% and 7.8%, respectively. The sensitivity of the insulin assay was 0.2 ng/mL and the intra- and interassay CVs were 4.9% and 7.7%, respectively. The sensitivity of the leptin assay was 0.6 ng/mL and the intra- and interassay CVs were 4.7% and 5.6%, respectively.

### Testicular ultrasonogram and pulsed-wave Doppler ultrasound examination

Testicular ultrasound evaluations were repeated every 28 days using a B-mode ultrasound scanner equipped with a 7.5-MHz linear array transducer (Mindray®, Z5 Vet, Shenzhen, China), as previously described (Rossi el al., 2019). The pixels intensity of images was analyzed as proposed by Ahmadi et al. (2013) using the Image Pro Plus 7.01 software (Media Cybernetics Inc., San Diego, CA, USA).

The mean diameter of the testicular artery was determined in the region of the spermatic cord of both testicles, as proposed by Wood et al. (2010) and Feliciano et al. (2012). This assessment was performed with a Z5 Vet color ultrasound system (Mindray®) coupled to a 7.5-MHz linear array transducer. The presence and type of blood flow was assessed in the testicles with Color-flow Doppler and the following parameters were evaluated: diastolic velocity (EDV), peak systolic velocity (PSV), resistive index [RI = (PSV - EDV)/PSV], and pulsatility index [PUI = (PSV - EDV)/mean velocity between PVS and EDV]. All analyses were performed as previously described (Rossi et al., 2019).

### Semen collection and evaluation

Semen collection was performed at 28-day intervals with the aid of an Autojac electroejaculator (Neovet®, Uberaba, Brazil). Andrological parameters were assessed according to the recommendations of the Brazilian College of Animal Reproduction (CBRA in the Portuguese acronym) (CBRA, 2013). Sperm concentration was estimated by spectrophotometry (SDM1, Minitube®, Germany). Sperm morphology was assessed according to the methodology proposed by Blom (1973). Sperm cells (*n* = 200 cells counted per bull, at each collection) were evaluated under a microscope equipped with differential phase interference contrast (Eclipse Ni-U, Nikon®, Tokyo, Japan) and were classified into major, minor and total sperm defects (CBRA 2013).

Scrotal circumference was measured at the time of semen collection with a scrotal measuring tape (CBRA, 2013).

### Semen cryopreservation

Semen was collected at 28-day intervals, but only the semen from the last collection was cryopreserved (a single batch per bull). The semen of 12 animals (6 low RFI and 6 high RFI bulls) of each replicate (year) of the experiment was frozen. Thus, considering that the experiment was repeated with different animals in 2 consecutive years, the semen of a total of 24 bulls was cryopreserved (12 low RFI and 12 high RFI bulls). The selection of bulls for sperm cryopreservation was based on the results of the previous andrological examination, being chosen only the bulls that showed the best seminal quality based on the following parameters: total motility ≥60%, vigor ≥3, major defects ≤10%, total defects ≤30% and concentration ≥350×10^6^ spermatozoa/mL [as recommended by CBRA (2013).

The collected semen samples were diluted in BotuBov® single-fraction diluent (BotuPharma®, Botucatu, Brazil) to a final concentration of 50×10^6^ spermatozoa/mL. Samples were loaded into 0.5-mL straws at room temperature and subsequently cooled and frozen in a programmable semen cryopreservation system (TK® 4000, Uberaba, Brazil). The cooling rate was adjusted to -0.25^o^C/min from room temperature (25^o^C) until a temperature of 5^o^C was reached. The straws were kept at 5^o^C for 4 hours for stabilization and then frozen at -20^o^C/min until a temperature of -120^o^C was reached. Next, the straws were directly transferred to liquid nitrogen (-196^o^C).

### Computer-assisted sperm analysis (CASA)

Sperm kinetic parameters were evaluated in fresh and frozen semen immediately after thawing two straws from each bull at 37^o^C for 30 seconds. The frozen/thawed semen samples were also analyzed by the CASA technique after submitting the samples to the rapid thermoresistance test (RTT) at 46^o^C for 30 min (Barnabé et al., 1980; Vianna et al., 2009). The semen samples were analyzed in an IVOS analyzer (version 14.0, Hamilton Thorne Biosciences, Beverly, USA) as described by Campanholi et al. (2017) and the CASA setup was preadjusted as described by Rossi et al. (2019).

### Flow cytometry

Frozen/thawed semen samples were analyzed by flow cytometry after incubation for 15 min at 37^o^C and again after incubation for 1 hour at 37^o^C (RTT). Mitochondrial membrane potential, production of superoxide in the mitochondrial matrix and plasma and acrosome membrane integrity were evaluated as previously described (Freitas-Dell’aqua et al., 2012; Freitas-Dell’aqua et al., 2016). The analyses were performed in a BD LSR II flow cytometer (Becton Dickinson, Mountain View, CA, USA) equipped with violet (405 nm, 100 mW), blue (488 nm, 100 mW) and red (640 nm, 40 mW) lasers. Equipment settings and adjustments were configured as previously described in Freitas-Dell’aqua et al. (2012). Data were analyzed using the BD FACSDiva v6.1 software.

### In vitro maturation, fertilization and embryo culture (IVP)

Cleavage and blastocyst production rates were the parameter used to determine the *in vitro* fertilizing ability of sperm. Ovaries were obtained from a commercial slaughterhouse. Follicles measuring between 2 and 8 mm in diameter were aspirated with the aid of a needle attached to a syringe and the cumulus-oocyte complexes (COCs) were morphologically evaluated under a stereomicroscope. The COCs surrounded by at least four layers of compact cumulus cells and homogenous granulated ooplasm were selected for the experiment. Selected COCs (*n* = 5,014, distributed among 6 replicates) were evenly distributed between experimental groups and matured as previously described (Kipper et al., 2017; Rossi et al., 2019). For *in vitro* fertilization, individual semen samples from all animals selected for semen cryopreservation were used (24 bulls in total, considering the 2 years of the experiment: 12 of which classified as low RFI and 12 classified as high RFI). In each year of the experiment, the semen straws of the 12 bulls selected in the year were simultaneously thawed at 37°C for 30 s and the content was centrifuged separately on a discontinuous Percoll density gradient (GE Healthcare, Bio-Science, Uppsala, Sweden) for 7 min at 2300*g*. The pellet containing the spermatozoa was resuspended in fertilization medium [as previously described in Kipper et al. (2017) and Rossi et al. (2019)] to obtain a final concentration of 2×10^6^ cells/ml. Then, 4 μl of the sperm suspension of each individual semen sample of the experiment were added to each fertilization droplet containing the matured oocytes (20-25 per 90-μl droplet). Oocytes and sperm were co-incubated for 18 hours, under the same temperature and atmosphere conditions used for IVM. All IVP procedures were performed following the steps described by Kipper et al. (2017) and Rossi et al. (2019). Cleavage and blastocyst development rates were assessed, respectively, 48 hours post-insemination (day 2) and 168 hours post-insemination (day 7).

### Statistical analysis

The results were submitted to analysis of variance for repeated measures over time using the PROC MIXED procedure of the Statistical Analysis System 9.2 software (SAS Institute, Inc., Cary, NC, USA). Means were fitted by the least squares method (LSMEANS) and compared, if necessary, by the probability of difference (PDIFF) using the t-test. The statistical model was: Y = µ + year + RFI + month + RFI*month + RFI*year + e, where y = variable; μ = overall mean; year = effect of year (i = 1, 2); RFI = effect of residual feed intake (i = 1, 2); month = effect of sampling time (i = 10 months); RFI*month = residual feed intake x month interaction; RFI*year = residual feed intake x year interaction, and e = error.

The statistical model for the flow cytometry and computer-assisted analysis of frozen/thawed semen was: Y = µ + year + RFI + RTT + RFI*RTT + RFI*year + e, where y = variable; μ = overall mean; year = effect of year (i = 1, 2); RFI = effect of residual feed intake (i = 1, 2); RTT = effect of rapid thermoresistance test (i = 1, 2); RFI*RTT = residual feed intake x rapid thermoresistance test interaction; RFI*year = residual feed intake x year interaction, and e = error

In these models, residual (co)variances were modeled to determine the structure of the repeated measurements. The lowest values for the Akaike information criterion (AIC) and Bayesian information criterion (BIC) were used to choose the structure of the (co)variance matrix that resulted in the best fit of the model.

Pearson correlation coefficients were calculated using the R software (version 3.6). The level of significance was set at *P* < 0.05. A *P*-value between 0.051 and 0.1 was considered a tendency.

## Results

### Feed intake and growth traits of the animals

Feed intake and ultrasound carcass parameters were not affected by RFI (*P* > 0.05). However, initial, final and average body weight, as well as hip height were higher in low RFI bulls (*P* < 0.05) compared to high RFI animals (Table 1). As expected, an effect of sampling time (from 14 to 24 months of age: month effect) was observed in all of these parameters (*P* < 0.05).

**Table 1:** Feed intake and growth traits of young Nellore bulls with divergent feed efficiency.

	**RFI**		**SEM^3^**	***P*-value** **4**		
	**Low**	**High**		**RFI**	**Month**	RFI*Month
*Feed intake* ^1^						
DMI forage (kg/day)	6.95	7.31	0.20	0.22	<0.001	0.94
*Growth characteristics* *2*						
Initial age (months)	14.4	14.3	0.13	0.61	.	.
Final age (months)	24.68	24.58	0.13	0.61	.	.
Initial BW (kg)	398.08	380.95	5.43	0.03	.	.
Final BW (kg)	534.29	508.08	6.09	0.004	.	.
Average BW (kg)	489.96	466.81	5.83	0.01	<0.001	0.80
ADG (kg)	0.44	0.41	0.01	0.42	<0.001	0.75
BCS (1-9)	6.19	6.15	0.07	0.74	<0.001	0.71
HH (cm)	147.11	144.53	0.60	0.01	<0.001	0.16
REA (cm^2^)	67.91	67.89	0.75	0.98	<0.001	0.67
BFT (cm)	1.45	1.54	0.09	0.52	<0.001	0.14
RFT (cm)	4.05	4.25	0.14	0.33	<0.001	0.42

^1^DMI forage = forage dry matter intake. ^2^BW = body weight; ADG = average daily weight gain; BCS = body condition score; HH = hip height; REA = rib eye area; BFT = backfat thickness; RFT = rump fat thickness. ^3^SEM = standard error of least squares mean. ^4^RFI = residual feed intake effect; Month = sampling time effect (from 14 to 24 months of age); RFI*Month = RFI by Month effect.

RFI was not significantly correlated with initial body weight, DMI, ADG, initial measurements of rump fat thickness, backfat thickness and rib eye area or final measurement of rump fat thickness (*P* > 0.05). However, RFI was significantly correlated (*P* = 0.04) with final body weight (r = -0.31). Additionally, there was a tendency towards a correlation between RFI and final measurements of rib eye area (*P* = 0.07) and backfat thickness (*P* = 0.09). The correlation coefficients are summarized in Table 2.

**Table 2:** Correlation coefficient between residual feed intake (RFI) and measures of growth performance, blood metabolic hormones, and body composition in young Nellore bulls.

**Variable 1**	**Variable 2**	**Correlation**	***P*-value**
RFI	Initial body weight	-0.19	0.21
RFI	Final body weight	-0.31	0.04
RFI	Dry matter intake	0.23	0.14
RFI	Average daily weight gain	-0.08	0.61
RFI	Plasma insulin	-0.05	0.75
RFI	Plasma leptin	-0.11	0.47
RFI	Initial rib eye area	-0.22	0.15
RFI	Initial backfat thickness	0.12	0.43
RFI	Initial rump fat thickness	0.05	0.73
RFI	Final rib eye area	0.27	0.07
RFI	Final backfat thickness	0.26	0.09
RFI	Final rump fat thickness	-0.10	0.50

### Circulating concentrations of metabolites and hormones

Mean glucose concentration was affected by RFI (*P* = 0.05), sampling time (*P* < 0.001), and interaction (*P* = 0.01). Insulin concentration was lower (*P* < 0.05) in low RFI animals when compared to high RFI animals (8.42 vs. 10.72 mg/dL, respectively; Table 3). In contrast, mean leptin concentration was higher (*P* < 0.001) in low RFI bulls (13.83 vs. 12.06 ng/mL). Mean cholesterol, HDL and testosterone concentrations were not affected by RFI (*P* > 0.05). The circulating concentrations of all tested hormones and metabolites were affected (*P* < 0.05) by sampling time (Table 3).

**Table 3:** Circulating concentrations of metabolites and hormones of young Nellore bulls with divergent feed efficiency.

	**RFI**		**SEM** **2**	***P*-value** **3**		
	**Low**	**High**		**RFI**	**Month**	RFI*Month
Glucose (mg/dL)	77.53	83.38	2.07	0.05	<0.001	0.01
Cholesterol (mg/dL)	140.76	145.39	3.30	0.32	<0.001	0.96
HDL (mg/dL)1	62.47	62.74	1.78	0.91	<0.001	0.43
Insulin (µU/mL)	8.42	10.72	0.39	<0.001	<0.001	0.20
Leptin (ng/mL)	13.83	12.06	0.32	<0.001	<0.001	0.85
Testosterone (ng/mL)	11.28	11.47	0.60	0.80	<0.001	0.99

^1^HDL = high density lipoprotein. ^2^SEM = standard error of least squares mean. ^3^RFI = residual feed intake effect; Month = sampling time effect (from 14 to 24 months of age); RFI*Month = RFI by Month effect.

RFI was not significantly correlated (*P* > 0.05) with plasma levels of insulin, leptin (Table 2) or testosterone (Table 4).

**Table 4:** Correlation coefficient between residual feed intake (RFI) and reprodutive parameters in young Nellore bulls.

**Variable 1**	**Variable 2**	**Correlation**	***P*-value**
RFI	Plasma testosterone	-0.16	0.29
RFI	Scrotal circunference	-0.19	0.21
RFI	Testicular ultrasonogram pixel intensity	-0.01	0.96
RFI	Testicular artery diameter	-0.08	0.61
RFI	Sperm concentration in the ejaculate	-0.10	0.50
RFI	Total sperm defects	0.04	0.77
RFI	Parameters evaluated by CASA1 in fresh semen: TM	0.07	0.64
RFI	PM	0.10	0.50
RFI	RAP	0.10	0.51
RFI	ALH	-0.13	0.39
RFI	BCF	0.08	0.62
RFI	LIN	0.15	0.33
RFI	Blastocyst rate (IVP2)	0.09	0.69

^1^CASA = computer-assisted sperm analysis; TM = total motility; PM = progressive motility; RAP = rapid cells; ALH = amplitude of lateral head displacement; BCF = beat cross frequency; LIN = linearity. ^2^IVP = *in vitro* production of embryos.

### Testicular ultrasound analysis

Testicular ultrasonogram, perfusion parameters of the testicular artery and testicular artery diameter were not affected by RFI (*P* > 0.05). However, there was an effect of sampling time on all variables studied (*P* < 0.05). The results of the testicular ultrasound examination are summarized in Table 5.

**Table 5:** Testicular ultrasonographic characteristics of young Nellore bulls with divergent feed efficiency.

		**RFI**		**SEM** **3**	***P*-value** **4**		
		**Low**	**High**		**RFI**	**Month**	RFI*Month
*Doppler of the testicular artery* *1*							
Diameter (mm)		2.75	2.70	0.03	0.25	<0.001	0.33
PSV (cm/s)		16.05	15.54	0.32	0.27	<0.001	0.97
EDV (cm/s)		7.77	7.31	0.29	0.26	<0.001	0.78
RI		0.50	0.52	0.01	0.55	0.03	0.67
PI		0.70	0.72	0.03	0.67	0.02	0.79
*US testicular parenchyma* *2*							
Medium		93.16	93.00	5.07	0.98	<0.001	0.82
Max		139.76	140.11	6.40	0.97	<0.001	0.83
Min		48.59	48.07	1.33	0.78	<0.001	0.63
Heterogeneity		0.10	0.10	0.004	0.46	<0.001	0.98

^1^Diameter = mean diameter of the testicular artery; PSV = peak systolic velocity; EDV = diastolic velocity; RI = resistive index; PI = pulsatility index. ^2^Medium = average pixel levels; Max = maximum; Min = minimum; Heterogeneity = heterogeneity of pixels represented by the standard deviation of the image. ^3^SEM = standard error of least squares mean. ^4^RFI = residual feed intake effect; Month = sampling time effect (from 14 to 24 months of age); RFI*Month = RFI by Month effect.

There was no correlation between RFI and testicular ultrasonogram pixel intensity or testicular artery diameter (*P* > 0.05) (Table 4).

### Fresh semen characteristics and scrotal circumference

Scrotal circumference, sperm concentration, percentage of defects and CASA parameters analyzed in fresh semen were unaffected by RFI (*P* > 0.05; Table 6). As expected, the percentage of major defects and total defects decreased over time, while the percentages of total motility (TM, %), progressive motility (PM, %) and rapid cells (RAP, %) assessed by CASA and the scrotal circumference increased (*P* < 0.05; Table 6). There was a RFI by month interaction (*P* < 0.05) for straightness (STR, %) evaluated by CASA, which was lower in the high RIF group in the summer season compared to the low RIF group (data not shown graphically).

**Table 6:** Reproductive characteristics and computer assisted-analysis of fresh semen collected from young Nellore bulls with divergent feed efficiency.

	**RFI**		**SEM** **3**	***P*-value** **4**		
	**Low**	**High**		**RFI**	**Month**	RFI*Month
*Mean variables* *1*						
SC (cm)	31.42	30.43	0.42	0.10	<0.001	0.65
Conc (x 10^6^/ml)	384.04	307.01	39.99	0.14	<0.001	0.79
Major Def. (%)	24.91	28.81	3.63	0.46	<0.001	0.81
Total Def. (%)	34.66	38.59	2.85	0.33	<0.001	0.95
*CASA* *2*						
TM (%)	76.43	71.31	2.2	0.11	<0.001	0.19
PM (%)	58.72	55.28	2.07	0.25	<0.001	0.18
RAP (%)	71.90	67.29	2.24	0.16	<0.001	0.15
VAP (µm/s)	87.58	86.89	1.58	0.76	<0.001	0.13
VSL (µm/s)	73.41	72.61	1.28	0.66	<0.001	0.09
VCL (µm/s)	138.45	137.53	3.26	0.88	<0.001	0.12
ALH (µm)	5.50	5.38	1.17	0.62	<0.001	0.23
BCF (Hz)	33.43	32.68	0.44	0.19	<0.001	0.16
STR (%)	84.04	83.80	0.40	0.54	<0.001	0.04
LIN (%)	56.59	56.88	0.88	0.86	<0.001	0.19

^1^SC = scrotal circumference; Conc = sperm concentration; Major Def. = major sperm defects; Total Def. = total sperm defects. ^2^CASA = computer-assisted sperm analysis; TM = total motility; PM = progressive motility; RAP = rapid cells; VAP = average path velocity; VSL = straight-line velocity; VCL = curvilinear velocity; ALH = amplitude of lateral head displacement; BCF = beat cross frequency; STR = straightness; LIN = linearity. ^3^SEM = standard error of least squares mean. ^4^RFI = residual feed intake effect; Month = sampling time effect (from 14 to 24 months of age); RFI*Month = RFI by Month effect.

No correlation was observed between RFI and scrotal circumference, sperm concentration, percentage of total defects or TM, PM, RAP, amplitude of lateral head displacement (ALH, μm), beat cross frequency (BCF, Hz), and linearity (LIN, %) analyzed by CASA in fresh semen (*P* > 0.05) (Table 4).

### Characteristics and quality of frozen/thawed semen

No effect of RFI (*P* > 0.05) was found on the parameters evaluated by flow cytometry in thawed semen. However, the percentages of cells with high mitochondrial potential and presenting intact plasma and acrosome membranes were lower after RTT (RTT effect: *P* < 0.05), whereas the percentage of O_2_-positive cells was higher (*P* < 0.05). Data are summarized in Table 7.

**Table 7:** Flow cytometry and computer assisted-analysis of frozen/thawed semen collected from young Nellore bulls with divergent feed efficiency.

	**RFI**		**RFI**		**SEM** **3**	***P*-value** **4**		
	**Low**	**High**	**Low**	**High**		**RFI**	**RTT**	RFI*RTT
*Flow cytometry* *1*	After thawing		After RTT*					
IPAM (%)	45.85	47.67	37.61	36.71	3.56	0.89	0.01	0.70
O_2_ positive (%)	45.91	44.96	56.14	52.55	4.35	0.60	0.04	0.76
HMP (%)	29.31	31.26	23.20	23.48	2.77	0.68	0.01	0.76
*CASA* *2*	After thawing		After RTT**					
TM (%)	75.50	75.66	73.25	68.25	4.63	0.60	0.30	0.58
PM (%)	56.41	58.08	61.83	57.83	4.11	0.77	0.53	0.49
RAP (%)	71.50	71.58	70.08	64.58	4.66	0.56	0.37	0.55
VAP (µm/s)	87.38	82.76	76.22	71.40	2.27	0.04	<0.001	0.96
VSL (µm/s)	70.01	67.56	65.75	62.46	1.72	0.10	0.009	0.80
VCL (µm/s)	144.71	135.04	118.92	110.10	4.51	0.04	<0.001	0.92
ALH (µm)	5.99	5.71	4.92	4.61	0.20	0.17	<0.001	0.93
BCF (Hz)	26.25	26.25	28.05	26.31	1.04	0.40	0.37	0.40
STR (%)	81.16	82.50	86.66	87.91	1.04	0.22	<0.001	0.96
LIN (%)	51.91	53.58	58.33	58.75	1.22	0.41	<0.001	0.62

^1^IPAM = intact plasma and acrosome membrane; O_2_ positive = superoxide anion positive cells; HMP = high mitochondrial potential. ^2^CASA = computer-assisted sperm analysis; TM = total motility; PM = progressive motility; RAP = rapid cells; VAP = average path velocity; VSL = straight-line velocity; VCL = curvilinear velocity; ALH = amplitude of lateral head displacement; BCF = beat cross frequency; STR = straightness; LIN = linearity. *Rapid thermoresistance test (RTT) for flow cytometry analysis: 1h at 37^o^C; **RTT for CASA analysis: 30 min at 46^o^C. ^3^SEM = standard error of least squares mean. ^4^RFI = residual feed intake effect; RTT = effect of rapid thermoresistance test; SUP*TRT = *P*-value between RFI and RTT interactions.

The parameters average path velocity (VAP; 87.38 vs. 82.76 µm/s for low and high RFI bulls, respectively) and curvilinear velocity (VCL; 144.71 vs. 135.04 µm/s, respectively) evaluated by CASA were higher in thawed semen of low RFI bulls when compared to high RFI animals (*P* < 0.05). A tendency towards higher straight-line velocity (VSL, μm/s) was observed in thawed semen of low RFI bulls (70.01 vs. 67.56 µm/s for low and high RFI bulls, respectively; *P* = 0.10). An effect of RTT was observed (*P* < 0.05) for VAP, VSL, VCL and ALH (decreased after RTT), and for STR and LIN (increased after RTT), as demonstrated in Table 7.

### In vitro sperm fertilizing ability

The rates of cleavage and embryonic development to the blastocyst stage were unaffected by RFI (*P* > 0.05), as demonstrated in Table 8. Similarly, RFI was not significantly correlated (*P* > 0.05) with blastocyst yields (Table 4).

**Table 8:** *In vitro* embryos production (IVP) with semen collected from young Nellore bulls with divergent feed efficiency.

**Embryonic outcomes** **1**	**RFI**		SEM*	***P*-value**
	**Low**	**High**		
Total oocytes (N^o^)	208,31	205,00	3.02	0.45
Cleavage rate (%)	77.26	79.30	1.49	0.33
Blastocyst rate (%)	34.32	37.45	1.46	0.13

* SEM = standard error of the mean. Averages in the row followed by different letters are different by the least squares method (P < 0,05). ^1^Total oocytes = sum of the number of oocytes from the 6 replicates of this experiment; Cleavage rate = rate of cleaved oocytes; Blastocyst rate = rate of blastocyst production at D7.

## Discussion

In the present study, the classification of bulls according to RFI was performed in a feedlot performance test and the animals were then maintained in a grazing regime throughout the experimental period. By definition, more efficient bulls (low RFI) consume less feed and still achieve the same weight as less efficient animals (Arthur and Herd, 2008). However, we found that pasture-raised low RFI bulls had a DMI similar to that of high RFI bulls maintained under the same conditions. Although this result seems to be contrary to expectations, previous studies have reported a lower DMI of low RFI animals in the feedlot, while DMI was similar to that of high RFI animals when maintained on pasture (Oliveira et al., 2016; Fitzsimons et al., 2013; Lucila Sobrinho et al., 2011). Despite the similar intake, the low RFI bulls studied here had a greater hip height and higher initial and final weight, suggesting better growth performance of more feed efficient animals during the pubertal phase. These results might be explained by the greater feed utilization capacity of low RFI animals (Arthur and Herd, 2008). Indeed, the present results demonstrated a tendency towards a higher final body weight in low RFI bulls. Thus, our results therefore reinforce the understanding that more feed efficient animals have the potential to positively impact the economy of the meat production sector (Kennedy et al., 1993; Forbes, 2007) because of their better body growth performance without an increase in feed intake.

Although there was no difference in the ultrasound carcass parameters between animals of divergent feed efficiency classes, low RFI bulls tended to have lower final backfat thickness. This result agrees with previous studies showing that animals with low RFI exhibit lower body fat accumulation (Richardson et al., 2001). Thus, selection for RFI may alter body composition and this should be taken into consideration when selecting animals for better carcass finishing.

The differences in the growth measures of animals with divergent feed efficiency may be due to metabolic alterations, which were analyzed in this work by assessing the circulating concentrations of hormones and metabolites. Although DMI did not differ between animals, lower plasma concentrations of glucose and insulin were found in low RFI bulls. The lower glucose concentration could explain the reduction of insulinemia in low RFI animals since insulin secretion is primarily regulated by glucose levels in extracellular fluids (Cerasi, 1975). Previous reports have shown similar results, in which lower insulin concentrations were detected in low RFI steers (Richardson et al., 2004). The lower insulinemia may thus explain the trend towards lower fat deposition in low RFI animals since the action of insulin promotes a reduction of lipolysis and increase of lipogenesis in adipocytes (Hocquette et al., 1998).

The vast majority of studies available in the literature that evaluated the effect of divergent feed efficiency on circulating leptin levels were carried out in beef steers and heifers of European breeds (*Bos taurus taurus*), whose results indicate that more efficient cattle had lower leptin concentrations (Foote et al., 2016). As this hormone is produced mainly by adipocytes in proportion to fat mass (Ahima et al., 2000), lower concentrations of leptin are expected in low RFI animals, which have greater lean mass and less accumulation of body fat. However, our results were opposite to those of the literature, as higher circulating leptin concentrations were found in low RFI bulls. Interestingly, our findings were similar to those of a previous study (Mota et al., 2017) carried out with young bulls of the same subspecies evaluated in our work (Nellore - *Bos taurus indicus*), which detected increased circulating leptin levels and its gene expression in muscle in the more efficient animals compared to the less efficient ones. Similar to these authors (Mota et al., 2017), we also observed lower fat deposition in feed efficient animals, which can be explained by the action of hormones related to the regulation of energy metabolism, such as insulin and leptin (Zieba et al., 2005). Moreover, leptin is a predictor of body weight (Thomas et al., 2002), which would explain our observation that heavier animals (low RFI) exhibited higher circulating leptin concentrations.

The opposite results regarding circulating leptin levels observed in zebu animals compared to European breeds are quite intriguing, suggesting that there may be an effect of genetics on the regulation of metabolism. In fact, the difference between these subspecies in terms of fat deposition in the meat is already well known, as Nellore meat is leaner due to the lower accumulation of intramuscular fat (Martins et al., 2015). Taken together, these results reinforce the need for further studies to assess the effects of the interaction between different breeds of cattle and the divergence in feed efficiency on metabolic activity and body growth performance. Furthermore, our results suggest that the age of the animal is also an important factor to be considered when interpreting variations in plasma leptin levels, as we observed that circulating leptin concentrations were affected by sampling time (month effect). Knowing that leptin also influences animal reproduction (Zieba et al., 2005), an increase in its concentration is expected as the animal reaches puberty and sexual maturity (Díaz-Torga et al., 2001; Garcia et al., 2002).

The impact of cattle selection for feed efficiency on fertility has not yet been fully investigated. Some studies reported worsening of seminal quality in low RFI animals (Arthur et al., 2005; Basarab et al., 2007; Mu et al., 2016; Awda et al., 2013; Hafla et al., 2012), while others found no change in fertility measures (Fox et al., 2004; Ferreira Júnior et al., 2018). Similar to the latter studies, we observed no correlation between RFI and the reproductive parameters of young Nellore bulls evaluated. In addition, there was no difference in plasma testosterone concentrations, ultrasound evaluation of the reproductive system or the quality of fresh semen between animals with divergent feed efficiency. Only sampling time exerted an effect on the reproductive traits. This was expected since the improved sperm quality of fresh semen and increased testosterone concentrations are positively correlated with older age of bulls and proximity to sexual maturity (Post et al., 1987).

The evaluation of frozen/thawed semen showed slight improvement of sperm velocity parameters (VAP, VCL and VSL) in low RFI bulls. These parameters are important to determine the ability of sperm to follow a certain path (Contri et al., 2010) and are related to the process of fertilization both *in vivo* and *in vitro* (Verstegen et al., 2002). Furthermore, as sperm velocity positively affects fertility outcomes, higher VCL values have been associated with higher cleavage rates of oocytes fertilized *in vitro* (Hwang et al., 1999). However, in contrast to expectations, we observed no effect of improved sperm velocity on cleavage rates or embryo production. Similarly, we found no correlation between *in vitro* sperm fertilizing ability and RFI. Although the cleavage rate is the best *in vitro* parameter to express sperm fertilizing ability (Amann and Hammerstedt, 2002), it is possible that the improvement in seminal quality observed in the present study was too subtle to affect the production rates in the IVP, especially considering that only the bulls that presented the best seminal quality according to the recommendations of the CBRA (2013) were selected for use in IVP experiments. Furthermore, there is also a possibility that the lack of effect of improved semen quality on the *in vitro* fertilizing ability observed in the present study was due to the selection method that was used for the separation of motile sperm before IVF (Percoll). As this procedure selects only viable sperm, this may have masked any potential effect of improved semen quality on IVP outcomes, as previously suggested (Rossi et al., 2019).

The quality traits of frozen/thawed semen were reevaluated after its incubation at 46^o^C for 30 min (RTT). In general, worsening of the velocity parameters and ALH was observed, as well as a reduction in mitochondrial function and sperm membrane integrity and an increase in O_2_-positive cells. These alterations in seminal quality after the RTT were expected since spermatozoa experience conditions of high temperature over a prolonged period of time, accelerating sperm metabolism and resulting in an energy deficit (Vianna et al., 2009). However, no RFI by RTT interaction was observed, suggesting that no differences exist between semen collected from bulls with divergent feed intake regarding the preservation of semen quality after the RTT.

## Conclusions

The findings of the current study indicate significant differences in the growth performance and metabolism of young Nellore bulls with divergent feed efficiency raised on pasture. On the contrary, no appreciable differences were observed in a set of fertility-related parameters. Despite slight improvements in semen quality of bulls with low RFI compared to high RFI animals, the *in vitro* sperm fertilizing ability was unaffected. Therefore, selection of beef bulls for RFI can be performed, which will result in economic benefits by improving the growth performance of the animals without affecting reproductive parameters.
